# Veterans’ Preferences for Exchanging Information Using Veterans Affairs Health Information Technologies: Focus Group Results and Modeling Simulations

**DOI:** 10.2196/jmir.8614

**Published:** 2017-10-23

**Authors:** Jolie N Haun, Margeaux Chavez, Kim Nazi, Nicole Antinori, Christine Melillo, Bridget A Cotner, Wendy Hathaway, Ashley Cook, Nancy Wilck, Abigail Noonan

**Affiliations:** ^1^ HSR&D Center of Innovation on Disability and Rehabilitation Research James A. Haley VA Medical Center Tampa, FL United States; ^2^ Department of Community & Family Health College of Public Health University of South Florida Tampa, FL United States; ^3^ Veterans and Consumers Health Informatics Office Veterans Health Administration Department of Veterans Affairs Washington, DC United States; ^4^ Department of Anthropology University of South Florida Tampa, FL United States; ^5^ Office of Health Informatics Veterans Health Administration Department of Veterans Affairs Washington, DC United States

**Keywords:** communication, patient participation, quality improvement, health information technology, medical informatics, patient portal, personal health record, telehealth, kiosk, mhealth

## Abstract

**Background:**

The Department of Veterans Affairs (VA) has multiple health information technology (HIT) resources for veterans to support their health care management. These include a patient portal, VetLink Kiosks, mobile apps, and telehealth services. The veteran patient population has a variety of needs and preferences that can inform current VA HIT redesign efforts to meet consumer needs.

**Objective:**

This study aimed to describe veterans’ experiences using the current VA HIT and identify their vision for the future of an integrated VA HIT system.

**Methods:**

Two rounds of focus group interviews were conducted with a single cohort of 47 veterans and one female caregiver recruited from Bedford, Massachusetts, and Tampa, Florida. Focus group interviews included simulation modeling activities and a self-administered survey. This study also used an expert panel group to provide data and input throughout the study process. High-fidelity, interactive simulations were created and used to facilitate collection of qualitative data. The simulations were developed based on system requirements, data collected through operational efforts, and participants' reported preferences for using VA HIT. Pairwise comparison activities of HIT resources were conducted with both focus groups and the expert panel. Rapid iterative content analysis was used to analyze qualitative data. Descriptive statistics summarized quantitative data.

**Results:**

Data themes included (1) current use of VA HIT, (2) non-VA HIT use, and (3) preferences for future use of VA HIT. Data indicated that, although the Secure Messaging feature was often preferred, a full range of HIT options are needed. These data were then used to develop veteran-driven simulations that illustrate user needs and expectations when using a HIT system and services to access VA health care services.

**Conclusions:**

Patient participant redesign processes present critical opportunities for creating a human-centered design. Veterans value virtual health care options and prefer standardized, integrated, and synchronized user-friendly interface designs.

## Introduction

Patients often have busy schedules and competing priorities and want to control how and when they receive health services to meet their personal needs [1]. They often prefer to complete health-related tasks quickly and efficiently. In recognizing the needs of patients and their demand for convenient, continuous care, the Department of Veterans Affairs (VA) provides health information technology (HIT) resources that put veterans at the helm of their care. VA HIT complements traditional means of service delivery (eg, face-to-face, telephone, mail) and gives veterans the power to maximize the efficiency and convenience of their health care experience [2].

VA’s remotely accessible HIT systems and apps include the My HealtheVet (MHV) patient portal, VetLink Kiosks, mobile apps, and telehealth services [3]. VA HIT supports veterans and their informal caregivers as active and informed proactive partners in their health care [4]. These tools help users manage appointments, keep track of medications, log personal health journals, record personal health care information and health measurements (eg, diet, physical activity, vital signs), communicate with their health care team, and access their electronic health record (EHR) [5].

The VA has embraced the era of virtual health care delivery and initiated national efforts to redesign and reorganize HIT services. To ensure that HIT reflects veterans’ needs and supports their sustained use [5,6], the VA has leveraged human-centered design strategies [7]. The aim of this study was to provide a deeper understanding of veterans’ preferences for using HIT for managing chronic health conditions [8] and to inform VA HIT system design efforts. For the purposes of this paper, we focus on results from methods (ie, focus groups and pairwise comparison activities) that contributed to redesigning VA’s HIT systems and apps.

## Methods

This participatory study used mixed methods and included an expert panel and veteran participant focus groups. The study protocol has been previously published [8]. Expert panel members (EPMs) and veteran focus group participants provided descriptive information about VA and non-VA electronic health resources that veteran participants use for health care management. Pairwise comparison activities of HIT resources were conducted with both groups. Rapid iterative content analysis was used to analyze qualitative data. Descriptive statistics summarized quantitative data.

### Sample and Sampling

#### Expert Panel

Snowball sampling was used to identify VA providers, key operational representatives, and VA subject matter experts who could serve as EPMs. This study focused on VA HIT, so non-VA technologists were not included in the expert panel. Initial invitations were emailed to operational partners who were asked to represent their departments or technology-focused workgroups and to nominate other experts as needed to address gaps. EPMs participated in monthly meetings for 6 months. Their input, along with veteran participant data, led to the development of the VA HIT Systems Matrix. This novel tool describes the existing VA HIT system and identifies veteran participants’ vision for the future of an integrated VA HIT system. The VA HIT Systems Matrix was ultimately used to conduct a pairwise comparison activity [6].

#### Veteran Participant Sample

Purposive sampling yielded a sample pool for veteran participant recruitment efforts from two sites. We used administrative data to identify veterans who were registered for MHV, had completed the in-person process of authenticating their identity, and had opted to use Secure Messaging: 16,399 veterans in Tampa, Florida, and 1205 veterans in Bedford, Massachusetts. A greater number of veterans had registered for MHV and telehealth in Tampa than in Bedford, accounting for the difference in number of potential participants from each site. Next, we reviewed the list of potential veteran participants and identified 260 Tampa and 198 Bedford veterans who also used VA telehealth services. This ensured study participants had access to at least two forms of VA HIT resources.

All 458 potential veteran participants were contacted and screened using a structured questionnaire. The structured screening questionnaire included items to determine whether potential participants met study criteria, including age (≥35 years of age), the presence of at least two chronic comorbid conditions (eg, diabetes, high blood pressure, chronic obstructive pulmonary disease) and use of specific VA HIT resources (including MHV, kiosks, mobile apps, telehealth). This tool also helped researchers determine if potential participants were high- or low-volume users of VA HIT. High-volume VA HIT users were defined as those using two or more types of VA HIT at least once a month. Low-volume VA HIT users were defined as those using fewer than two VA HIT platforms less than once a month, and using two or more other electronic resources at least once a month.

We recruited approximately 10% of the sample pool. Ultimately, 47 veteran participants (44 male veterans and 3 female veterans) and one caregiver were grouped based on chronic health conditions and frequency of technology use (high, low). One female caregiver participated in a high-volume focus group. One female group (n=3) was convened to address woman’s health issues in addition to health conditions. This single group of females represented high-volume HIT users. Two other types of groups were formed: chronic conditions groups (n=7 groups) (eg, chronic obstructive pulmonary disease, diabetes mellitus, high blood pressure) and mental health groups (n=6 groups). These condition groups were then divided into high- and low-volume HIT use groups. See Table 1 for further details.

**Table 1:** Focus group composition.

Gender	User level	Group condition	Focus groups n	Total participants n
Women	High volume	Chronic condition	1	3
Men	High volume	Chronic condition	3	15^a^
Men	Low volume	Chronic condition	3	8
Men	High volume	PTSD and mental health	4	13
Men	Low volume	PTSD and mental health	2	9
Total			13	48

^a^Female caregiver participated in one high-volume chronic condition focus group.

### Data Collection and Asset Development

Data were collected in two phases. In Phase 1, 48 focus group participants described their current use of VA and non-VA HIT and modeled their preferences for using these technologies in the future. A 16-item focus group guide incorporated free-listing (listing items based on their knowledge) and simulation modeling activities [8]. During the first set of focus groups, veteran participants discussed VA HIT system access, design, and functionality preferences in relation to their specific health care management tasks (eg, refilling prescriptions) and identified their vision for the future of an integrated VA HIT system. These data informed development of the aforementioned VA HIT Systems Matrix. This Matrix is a large detailed inventory of virtual platforms, their features, and contexts for use. It has been previously published and is omitted from this publication [9]. The Matrix provides information on the patient-facing platforms that are available to veterans (eg, MHV, mobile health, kiosks, telehealth), key system features (eg, Secure Messaging, Blue Button), access/availability, user groups, and context of use. The Matrix was used as an informational tool that helped veteran participants and EPMs complete the analytical hierarchy pairwise comparison process activity in Phase 2 of the study, further described below.

**Figure 1:**
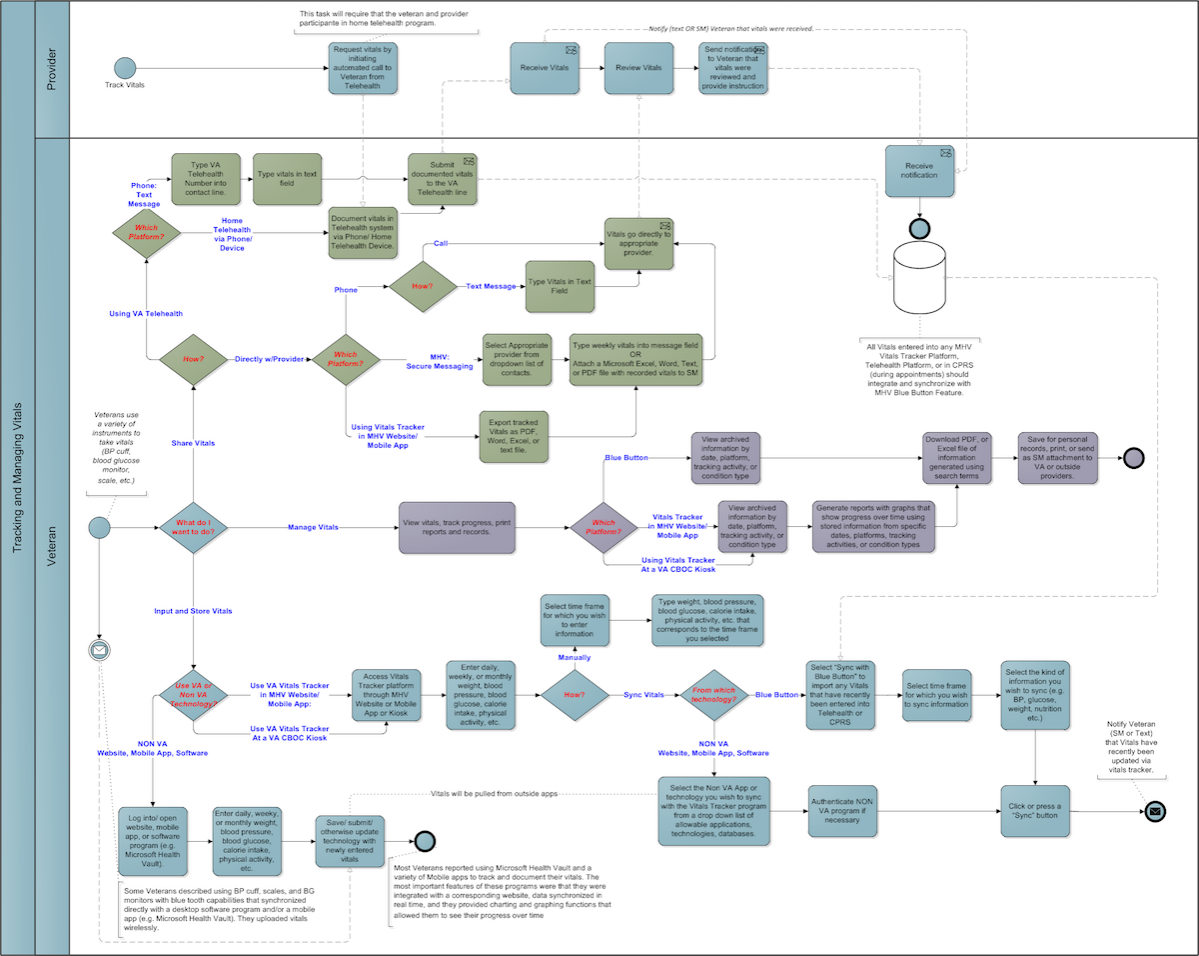
Process model example for tracking vitals.

Focus group data from Phase 1 also informed development of user personas, user scenarios, and low-fidelity representations (schemas, drawings, and process models) of participants’ system design and functionality preferences. User personas were “characters” developed to represent a veteran user in the scenarios. Process models provided a mapping strategy for developing interactive modeling simulations. A process model example is illustrated in Figure 1. The VA Human Factors Engineering (HFE) team used these assets, veteran comments provided through the MHV site, changes requested by VA clinicians, other veteran feedback provided by the VA Office of Connected Health, and an independent HFE study to create high fidelity, interactive, visual simulation models using iRise software [10]. HFE created the simulation with Structured Query Language (SQL) databases that enabled functionality similar to a live website. These functions included form submission, registration and credentialed sign-in with user recognition, live-updated data (dates or data previously submitted through forms), and validation error prompting. The simulation allowed a user to realistically use the prototype to support several representative veteran workflows such as refilling a prescription or canceling an appointment.

**Figure 2:**
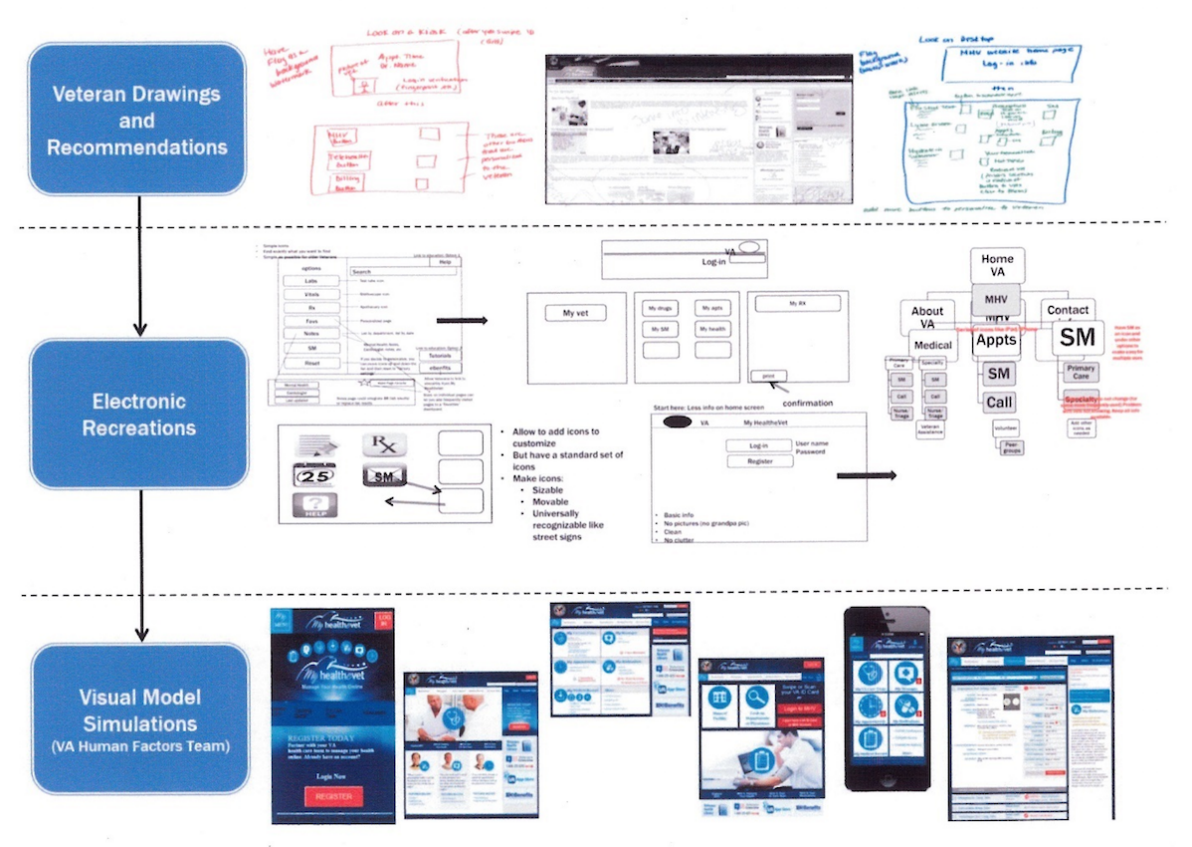
Process of creating simulated models.

The interactive simulations of redesigned VA HIT functioned on a variety of platforms (eg, mobile phone or tablet, desktop, kiosk) in test scenarios. These simulated models included mock app screens and webpages for platforms of interest (ie, Web, mobile, telehealth, kiosks). Participants provided feedback to refine modeled content in Phase 2 focus groups. This dynamic process of creating simulated models from participant data is illustrated in Figure 2.

**Figure 3:**
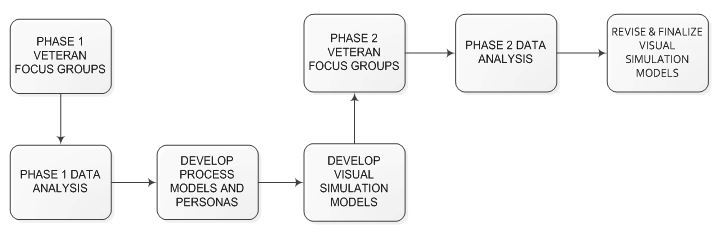
Data collection flow chart.

**Figure 4:**
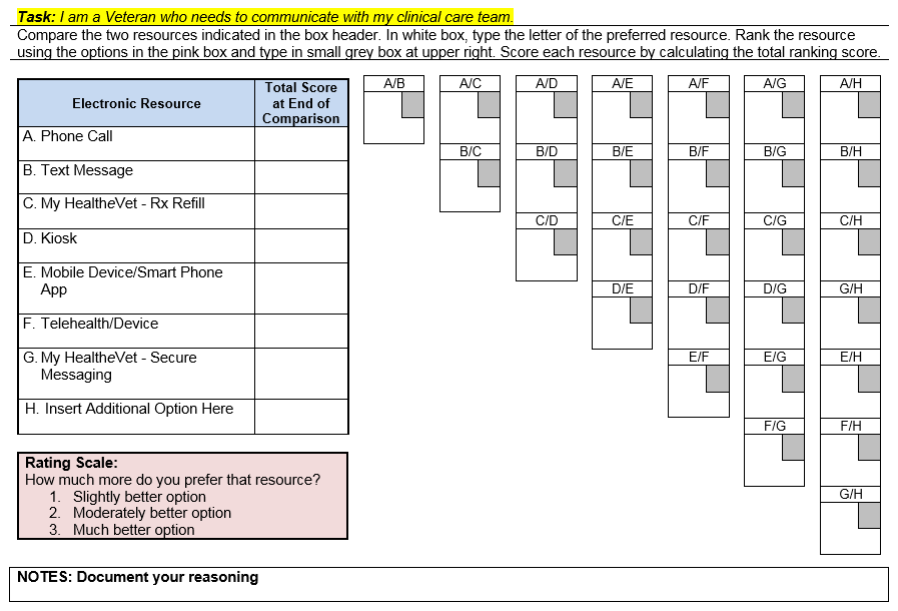
Sample page from pairwise comparison worksheet.

In Phase 2, participants from Phase 1 focus groups were divided into six Phase 2 focus groups based on participant availability. They reviewed the simulations of VA HIT and provided feedback on (1) accuracy of visual simulation models in capturing focus group input, (2) relevance of test scenarios, and (3) simulations’ design and functionality. Focus group facilitators gave a semi-scripted presentation that integrated veteran participant personas and user scenarios, multiple health management scenarios, and simulated prototypes of VA HIT on a variety of patient-facing platforms. Respondents’ reactions and experiences as they interacted with the simulations were audio recorded. Veteran participants also completed a similar pairwise comparison activity together at the end of each focus group. The data collection flow chart is illustrated in Figure 3. Participants then completed an analytical hierarchy pairwise comparison process activity [7]. This activity was conducted using a structured hierarchy of options for completing specific health care management tasks with VA HIT. The goal of the activity was for participants to select the best tool for completing a given task, by ranking alternatives. See the pairwise comparison worksheet in Figure 4.

### Data Management and Analysis

Focus group data were transcribed and managed using the qualitative data analysis software program ATLAS.ti version 7.1 (ATLAS.ti Scientific Software Development). Data were analyzed in two stages [11]. The first round of coding included summarizing and data reduction from notes and transcripts into preliminary metadomains. Methods included deductive, structural coding with codes derived from the interview guide, and inductive, descriptive coding with codes that emerged from the data. A second round of coding allowed researchers to reduce coded data into meaningful domains and themes. The research team established an interrater reliability rate of 80%.

## Results

Focus group participants represented a diverse veteran cohort and one caregiver who represented a veteran as a delegate. Participants were primarily male veterans with some college education, living with an average of six comorbid health conditions. Demographic data are presented in Table 2 and participant health conditions data in Table 3.

**Table 2:** Participant demographics (N=48).

Characteristics		n (%)
**Gender**		
	Female	4 (8)
	Male	44 (92)
**Status**		
	Veteran	47 (98)
	Caregiver	1 (2)
**Education**		
	High school	7 (15)
	Some college/vocational	20 (42)
	Associates degree	7 (15)
	College degree	7 (15)
	Graduate degree	7 (15)
**Race**		
	Caucasian/white	40 (83)
	African American/black	5 (10)
	Native Hawaiian/other Pacific Islander	1 (2)
	American Indian/Alaskan Native	1 (2)
	Other-American	1 (2)
**Ethnicity**		
	Hispanic or Latino	2 (4)
	Not Hispanic or Latino	45 (94)
	Declined to respond	1(2)
**Marital status**		
	Married	28 (58)
	Divorced	17 (35)
	Single/never married	3 (6)
**Annual income (USD)**		
	≤ $4,999	3 (6)
	$5,000-$10,000	1 (2)
	$10,001-$15,000	2 (4)
	$15,001-$25,000	7 (15)
	$25,001-$35,000	7 (15)
	$35,001-$45,000	6 (13)
	> $45,001	17 (35)
	Declined to respond	5 (10)

**Table 3:** Participants’ self-reported health conditions (N=48).

Health condition	n (%)
High blood pressure	35 (73)
Diabetes	27 (56)
PTSD/ Mental health	22 (46)
COPD/ Heart	22 (46)
Pain	11 (23)
Sleep disorder	9 (19)
High cholesterol	9 (19)
Any arthritis	7 (15)
Neuropathy	7 (15)
Cancer	6 (13)
Hearing problem	5 (10)
Hyperthyroidism	4 (8)
Kidney Issues	3 (6)
Acid reflux	3 (6)
Human immunodeficiency virus	2 (4)
Hernia	2 (4)
Gastroesophageal reflux disease	2 (4)
Headaches	2 (4)

### Focus Groups

#### Current Use of VA Health Information Technologies

All participants reported that electronic health tools and portals such as MHV and its component features are useful for managing health. Both types of user groups reported using (1) Secure Messaging (SM), a secure communication tool (like email) with VA health care providers, (2) Prescription Refills (Rx Refill), a secure online prescription refill program, and (3) MHV Appointments, an online resource that allows users to view past and future VA appointments as a list or on a customizable “Health Calendar” and to set up email reminders for upcoming appointments. High-volume HIT users were more likely to use telehealth, VA Mobile Apps, and additional MHV features, including the Blue Button, which allows veterans to view and download a copy of data from their EHRs, and the Veterans Health Library, an online veteran-focused library of reviewed health education resources. Sample quotes of current use of VA and non-VA HIT are included in Table 4.

**Table 4:** Sample quotes of current use of VA and non-VA HIT.

Domain		Theme	Sample quotes
**Current use of VA HIT**			
	General		I use My HealtheVet to manage appointments, to check on appointments, to look at lab results. I look at it to order prescriptions and check on my prescription refills to see what is available and what is left. When I get low on refills, I can contact [my care team] through [SM] to let the pharmacy and doctor know that I need to have something renewed.
	Use of the Secure Messaging		Secure Messaging is very helpful. I like the fact that if you have a question and you can’t get in [to the office] to see your primary care provider, at least you will get a nurse or whoever is on the other end giving you some information.
	Capabilities of Rx Refill for managing many prescriptions		I manage a lot of prescriptions, about 30 or 40 of them. Sometimes I get a new one and I use it for a month and then I don’t need it anymore. I can go on my [RX Refill] page and see what I’m taking… but [the page] still has drugs on there from 2 years ago that I’m no longer using. It’s hard to get the system to wipe them out and it can be really confusing.
	Function of MHV Appointments		[Those Appointments] are never up to date. Sometimes I get a call saying that I have an appointment scheduled for such and such a day at this time, but that will be the first I’ve heard of having an appointment. Those calls don’t say what appointments you have that day, they just say you have one. So, I go online to my calendar and, sure enough, there is nothing [indicating I have an appointment]. So, I don’t go. Turns out I did have an appointment that day and I get dinged on my record.
	Function of Blue Button		They have an item called Blue Button and on the Blue Button you can determine what information you want from your records. For example, lab results. You can [enter] a date range and say, “I want these items.” It has got a full checklist. You check those items and [Blue Button] will give you a full report. You can download the report as a PDF and review.
	Availability and utility of VetLink Kiosks		Kiosks? We don’t have those here, but I used one in New York to check in [to an appointment] at the hospital. I didn’t have to wait at the desk and someone was showing us how to use it. I’d like it if I could print my prescription list before my appointment, but maybe that would bring up [privacy] issues because the kiosk is right in the lobby.
	Utility of telehealth as a tool for attending therapy		I go for therapy through telehealth. The therapist is [at the hospital], I’m in [my location], and it’s incredible. It is so realistic that when I’m done and I get up and just walk out, [I feel] like I should shake his hand. [Using telehealth], I have a [therapy] group, and I have [one-on-one therapy] and then I have a third [therapy] with my psychiatrist for the medication.
	Creating VA Mobile Apps for health care management		I use my [tablet] for everything, but I can’t [access] the My HealtheVet [website] there. You can only access it on an actual computer or laptop so that’s why I was saying maybe they can come up with an app where you can access [My HealtheVet] from other places other than just the home computer because sometimes you’re out and you don’t have a way of getting any information until you get back to your house.
	Telephone		I’ll call the nurse when I need a prescription renewed. I like SM for questions and prescriptions too, but sometimes you just want to make that call.
**Non-VA HIT Use**			
	General		With Google, you don’t have to really look hard to find something, it’s pretty much right there in front of you. If you put [a topic] in your search bar, you are going get the [results] you are looking for. [My HealtheVet] is very difficult to manipulate because you have to figure out how to just get [to the search bar].
	Chronic health conditions		My daughter got me [a Fitbit] for Christmas because I needed to lose a lot of weight. I’ve lost 70 lbs since my operation…and that Fitbit has done it. I just got my 500 mile award the other day.
	Personal health information management		I’ve got high blood pressure and diabetes, so I have to check blood sugar levels and monitor my pressure every day. My BP cuff and my glucose monitor both have bluetooth so I just link them up with my [fitness] app and the information goes right in. It’s great for me because I can just pull out my phone when I see the doc and show him all the graphs and charts with my data.

#### My HealtheVet

The most commonly used resource by participants was MHV, particularly the SM and Rx Refill tools. Participants valued SM to communicate with their health care teams. Frequent communication included lab management, appointments, medications, general health concerns, and specialty care requests. Many participants preferred SM because it maintains a record of their communication. High- and low-volume HIT user groups agreed SM was easy to use because it mimicked familiar email formats. They appreciated SM’s convenience, stating that providers responded quickly, and veterans could better manage their care while avoiding telephone waits or travel to their local VA facility. Barriers to using SM included providers who were not active SM users, perception of usability issues (ie, too many steps required to log in), and being less convenient than using personal email.

Most participants liked the convenience of ordering prescription medications through Rx Refill. They requested refills in advance and could print Rx Refill pages for their personal records and community (non-VA) providers. High- and low-volume HIT users felt Rx Refill was complicated and did not adequately support management of several prescriptions. For example, users wanted notification when a prescription was going to expire, rather than scrolling through multiple online pages searching for refills or renewals. Many participants felt their Rx Refill page was cluttered with out-of-date prescriptions that impaired their ability to easily review current medications.

Most participants used MHV Appointments, including reminders and a health calendar to check for future appointments and look up appointment instructions. They indicated this tool was not always current, and appointment notifications were often updated late or not at all. They suggested adding details about appointment location (eg, unit, room, floor) and accessible details about past appointments. Veteran participants were concerned that information they provided through the MHV Appointments platform was rarely relayed in a timely fashion to their care teams.

Participants, particularly high-volume HIT users, reported using the Blue Button feature to print labs for community providers and personal records. Some participants reported difficulty using Blue Button, especially when accessing and interpreting lab results, and many felt that there were often too many pages to print out. Last, the Veterans Health Library was used by only one veteran participant in this study. Most veteran participants preferred easy to use non-VA sites for medical information (eg, WebMD).

**Figure 5:**
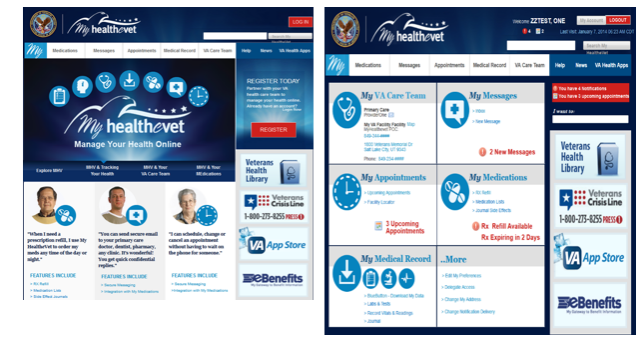
MHV home page and dashboard simulations.

In general participants reported a desire for clean dashboard designs that were user friendly and easy to navigate. Modeling simulations that were prepared based on veteran participant‒reported preferences for the MHV home page and dashboard are illustrated in Figure 5.

#### VetLink Kiosks

At the time of the study, VetLink Kiosks were not widely available at the study sites. A kiosk is a veteran-facing touch screen device, found in VA clinics, that allows veterans to perform basic tasks such as checking into an appointment. Some participants used kiosks at appointment check-in, but believed kiosks had additional potential. Participants envisioned using kiosks to view their entire integrated EHR, search their medical records, and print information. They wanted the ability to print facility maps. They conceded privacy risks associated with accessing this information in view of the waiting room and suggested building a cubicle around kiosks to provide privacy.

**Figure 6:**
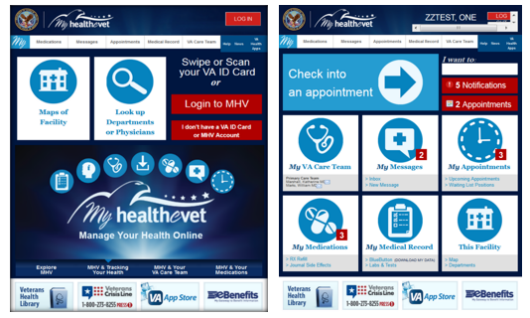
VetLink Kiosk simulations.

Veteran participants reported a desire for kiosks to be standardized, synchronized, and integrated with other VA HIT, particularly MHV. They reiterated their desire for a clean, user-friendly design. Modeling simulations that were prepared based on veteran participant‒reported preferences for the VetLink Kiosks are illustrated in Figure 6.

#### Telehealth

Veteran participants reported that telehealth services improved access to care. Veteran participants who used telehealth frequently used home telehealth to send vital signs to providers; however, they did not have access to previous submissions, making the tool ineffective for personal health monitoring. Participants reported preferring older telehealth equipment to the newer models of the phone telehealth system because the phone was too time consuming, though they did not provide specific details. A minority of veteran participants used video telehealth to communicate with providers for speech pathology and therapy appointments.

**Figure 7:**
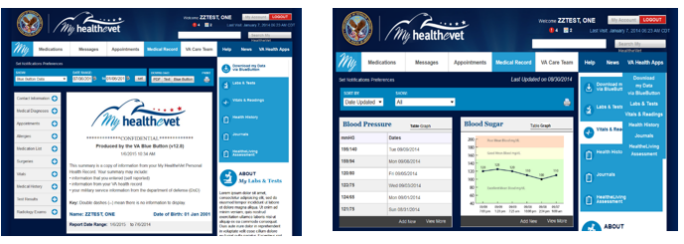
Simulations of “Medical Record” containing Blue Button and Vitals/Readings features within My HealtheVet.

The primary theme that emerged was veteran participants’ preference for synchronization, integration, and access to their data, particularly through MHV and Blue Button. Modeling simulations prepared using veteran participant‒reported preferences for access to their vital sign data are illustrated in Figure 7.

#### Mobile Apps

Few veteran participants used VA Mobile Apps, often due to reports of limited awareness of the available apps. It is also important to note a limited number of apps were available during this study, though many were in development and of interest to this study effort. There was a desire for convenient and easy-to-use apps. For those reporting use of the apps, Post-Traumatic Stress Disorder (PTSD) Coach, was most often cited, albeit infrequently. Veteran participants reported wanting mobile SM, appointment reminders, and Rx Refill apps or a single MHV app that integrated and synchronized all these features. Mobile apps preferences stemmed from a desire to have all health care management platforms conveniently located in a single place. Many veteran participants, particularly those with mental health issues, stressed the importance of creating secure mobile technologies.

#### Telephone and Mobile Phone

**Figure 8:**
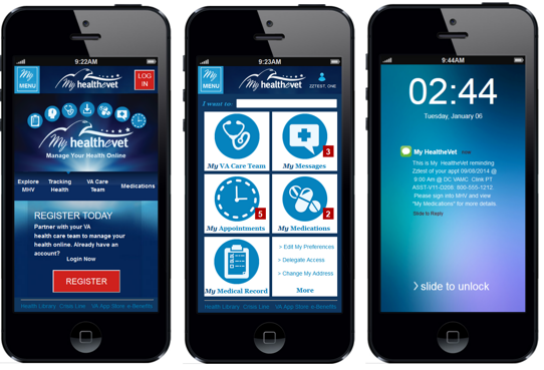
Modeling simulations of mobile phone designs and text features.

Participants used the telephone and mobile phones to supplement online activities. They communicate with providers, request prescription refills, and manage appointments. Phone use depended on status, urgency, and the level of accountability they wanted for a given issue. Participants reported a strong preference for using mobile phone technology to access MHV, mobile apps, and text alerts. Participants felt that although text messaging is not secure, there are appropriate uses for this technology such as appointment reminders and medication notifications. Modeling simulations for mobile phone designs and text features based on veteran participant preferences are illustrated in Figure 8.

### Current Use of Non-VA Health Information Technologies

High- and low-volume HIT users used Web browsers and search engines. Both groups preferred “clean,” “intuitive,” “simple to use” search engines that provided quick results. Participants with chronic health conditions used non-VA health technologies (eg, wearable heart, sleep monitors, pedometers) to better manage their condition. They used non-VA mobile apps to accomplish personal tasks such as tracking health parameters (eg, vital signs, weight, sleep patterns). These programs were described as “purposeful” and “tailored” to specific conditions and needs. High- and low-volume HIT users used multiple devices (eg, desktop, tablet, phone) noting the importance of quickly connecting to, and synchronizing information, across devices.

### Preferences for Future Use of VA Health Information Technologies

#### Electronic Communications

Veteran participants preferred exchanging information with providers electronically. Participants placed value on the use of SM to generate a record of communication that is accessible and accountable. They conceded that physician response time and adoption of this communication tool varied, and that the VA needs to implement mechanisms to overcome these barriers and improve SM effectiveness. Text messaging was thought to be the next logical platform for communicating with care teams. These tools would maintain the immediacy of a phone call and provide accountability by establishing a record of interaction. Sample quotes for future use of VA HIT are included in Table 5.

**Table 5:** Sample quotes of preferences for future use of VA HITs.

Theme	Sample quotes
On using electronic communications with providers	I’ve noticed that SM can be hit or miss. I’ve got some doctors who really use the thing. They get back to you right away and it’s great, but if your doctor doesn’t use SM then you are relying on the phone or going in to the hospital. [SM] is a great service as long as your doctor is using it.
Notifications or alerts	I have reminders coming in via emails, via text and all I have to do is hit accept and it goes on the calendar in my iPad. If it was that simple with the VA, I would be reminded of every appointment and they’d never have to send out another piece of mail again, the VA could save all this money on sending me these [appointment reminder] cards.
	They could communicate a lot of stuff to the vets through My HealtheVet. Every time you log on [the Vet could] have a [notification] message. It could be anything. It could be “we’re having a special on blood tests this week” or “your next appointment is [pause].” Could be tons of things they could put in there.
System integration and synchronization	I would like to have all [VA technologies] linked together in one place and that’s why I’ve been using the [Microsoft] Health Vault. If [the VA] could combine telehealth with My HealtheVet that would be the best website you could go to but also make the information available.
	I travel and [prefer] not having to be tied to a home computer. Anywhere we are with a tablet or phone, we could find out our information, our appointments, our medications, lab work, all the things we need would be available where ever we are whether I’m in an RV driving to the Grand Canyon or whether I’m at home or even in Europe where I could still do it with a mobile app.
Standardization	I think they should all be very similar, same similar appearance anyway. They don’t have to be the same but give me the same appearance where if it says Blue Button on one, it says Blue Button on another. If it was set up like Microsoft in your windows where I don’t care if you use your phone, your laptop or your home computer when you turn it on, you’re going to see the same thing every time. Like you said different items in different locations, but they’re all the same items and all the same design and the same look.
Design	I’m saying it should be something simple that if I went and opened the program up, whether it be a button, a little logo, whatever it’s going to have, something that would say, be in the shape of a needle I need immunizations…click, something simple that I could identify each thing that I’m going to look for. Use the “KISS” method…”keep it simple…”
	I normally now go to my Windows 8.1; it has a completely different look to it. It’s simple, it’s pictures and letters, and it tells you. for example, I look at this and I go this is my email, this is my contact list, this is my…and we can do the same for the VA…this is my medication, this is my appointments. I want little boxes, windows to tell me where to go.
Authentication	And whether you get it on the identification card, the microchip which will keep track of that or however, but one time you do need a face to face with somebody to verify who you are who you say you are.
	Why not online like the bank, banking online. You just sign up, you put in your security questions, whatever they ask you and then they send you back a confirmation email.
Delegation and sharing information with community providers	I want to be able to send my outside and VA provider an email with my records of my meds or labs or surgeries, but securely. I don’t want to have to go here and there requesting my records. It’d be great to give outside providers limited or one-time access to your records so they could see your [medical] history.
	My brother picks up my laptop and gets on My HealtheVet and he starts ordering stuff for me; technically that should not be allowed because I didn’t authenticate him. But if at the same time, I say to my brother I’m in bed, I can’t do it, can you go to my computer; there should be a method where I should be able to let him do that for me.
	You would have to be able to give your permission and once you give your permission they should have access. If I’m going to be an invalid and I can’t make decisions for myself like turning the power of attorney over to someone, they should have access to everything I have access to.
Single sign-on for federated credentialing	I think if you’re a vet, there’s difficulty in maintaining what your passwords are sometimes, guys lose them and they don’t remember, I think there’s merit in having just one login. The downside on the fact that I work with websites and that is that you do expose security cause if somebody gets the one they’re going get everything.
	Now the VA is using all the other federal agencies to get information on a veteran–they have access to my social security, they have access to my IRS information, my 1010 that I got for benefits‒so I don’t have a problem with one password being utilized after I [have] vetted with the VA to make sure I am who I [say] am. I don’t want to have to do a separate [password] for eBenefits or social security…or whatever other government agency I deal with…it should be all one.
Accessing information and education about VA HIT	I think if the VA really wanted to, there should be opportunity or classes, hey we’ll sit in a conference room with a big screen and I’m not trying to create a job for me or anybody else, but get a guy that’s a novice like myself and say okay, “hey guys let me show you this website, this is how you get to it, this is how you use”…And I think it should be another veteran, I think it needs to be somebody who is just a layman who says we’re going to go through My HealtheVet and just make that person comfortable.

### System Design Preferences

Participants drew inspiration from their personal use of technology and each other to model system design, functionality, and features. Models were created using large notepads, paper, and markers to demonstrate their preferences for system integration and synchronization. These data findings are described in the following sections.

#### Access, Presentation, and Navigation

**Figure 9:**
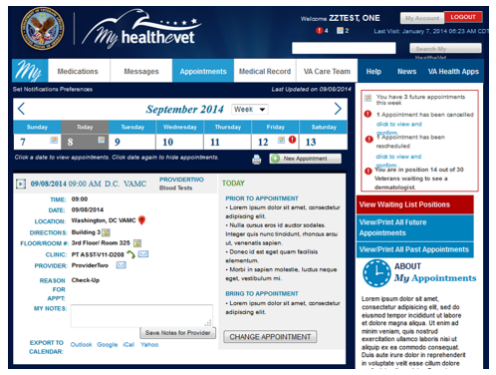
Modeled simulation of My HealtheVet appointment manager.

A salient theme from the focus groups was participants’ desire for notifications and alerts. They reported preferences for notifications when secure messages are sent/received; appointments are made/changed; prescriptions are refilled, adjusted, added, or expired; labs are ordered or results are available; and progress notes are available. Participants felt strongly that incorporating text message, phone-based, or SM notifications into the appointment reminders platform was important to facilitate patient appointment adherence. For example, text messaging was the most desirable platform for receiving notifications. Participants also felt notifications via SM, phone, mail, and personal email accounts would be acceptable and beneficial. Figure 9 illustrates a modeled simulation of an MHV appointment manager based on participant-reported preferences.

**Figure 10:**
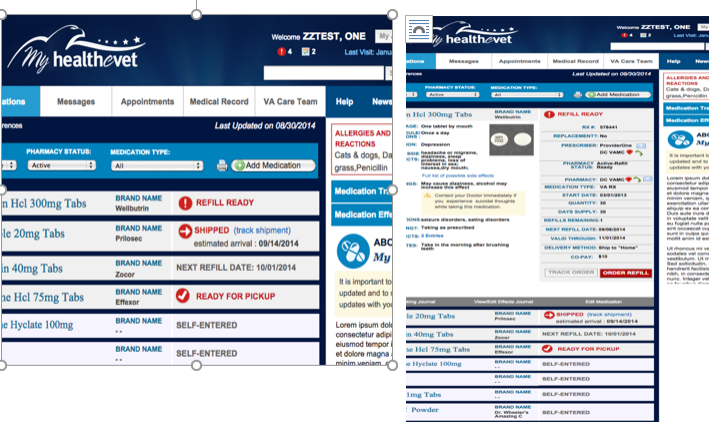
My HealtheVet Medication Manager in collapsed and expanded view.

Medication management was also a top priority for participants. They had clear expectations for a user-friendly system that allowed management of many medications. Participants voiced a strong preference for medication lists that could be easily collapsed and expanded for managing information quickly and efficiently. Modeling simulations of this collapsing and expanding medication management system are illustrated in Figure 10.

**Figure 11:**
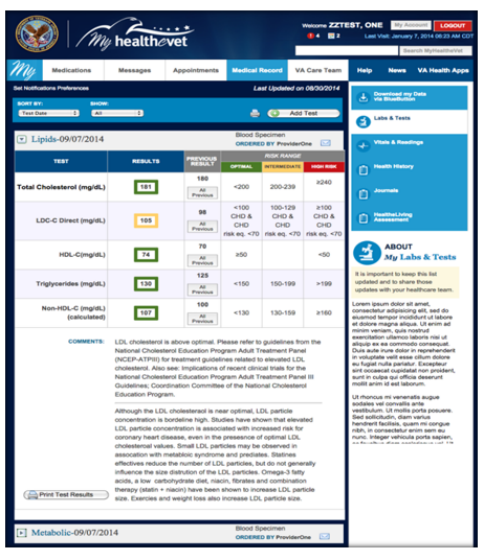
My HealtheVet Labs & Tests feature.

Last, participants wanted notifications and increased ease of access to lab results. Participants also reported problems interpreting lab results, voicing a strong preference for results to be illustrated in a user-friendly format with graphs and imagery. An example of a simulation based on their preference is presented in Figure 11.

#### System Integration and Synchronization

**Figure 12:**
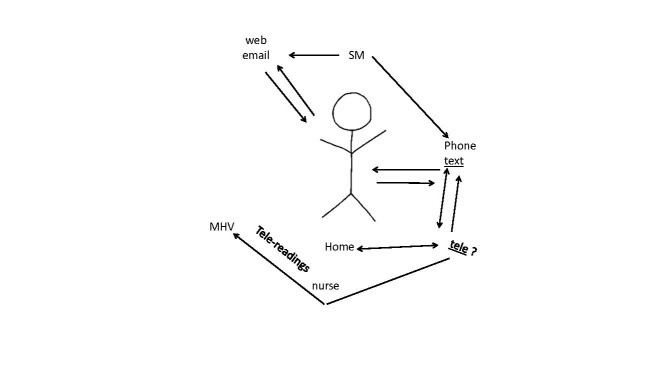
Veterans’ drawing of system design preferences.

Participants reported a strong preference for all of their health information to be synchronized, integrated into their EHR, and accessible to them online. They desired changes to their electronic medical record to update within hours and be rapidly accessible. For example, a participant drew an image indicating a need for all HIT to be integrated, to exchange information provided by and to the patient across systems. Participant renderings were re-created to illustrate their preferences for system functionality (see Figure 12).

**Figure 13:**
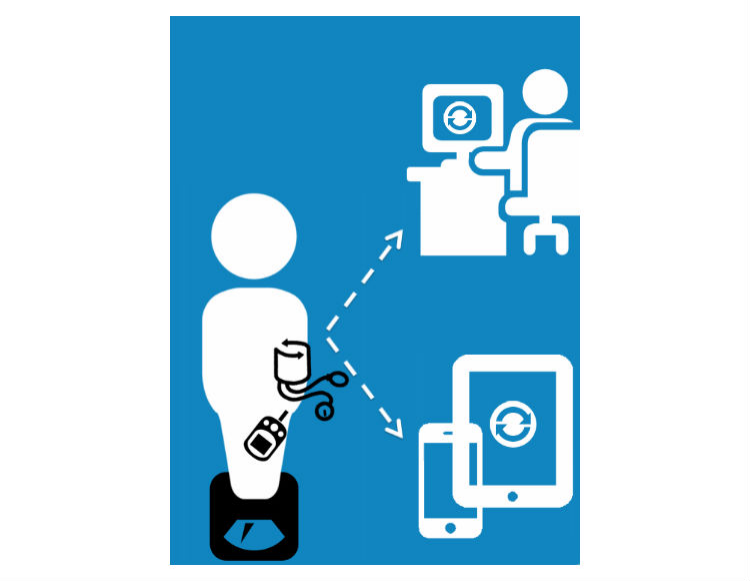
Participant conceptualization of synchronized vitals between VA and personal software programs using telehealth technology.

Some participants felt kiosks should also provide access to MHV records. In general, they felt synchronization and integration would significantly improve their health care management experience, particularly when managing appointments, medications, and vital signs. Figure 13 provides a conceptualization of their reported preference for providing vital sign information to their VA provider while also being able to immediately access and store that data on their personal software programs for self-care management.

#### Standardization

Veteran participants felt that standardizing the look, feel, layout, and navigation of all VA tools and platforms would make learning to use different technologies easier for diverse audiences. Participants also voiced preferences for universally recognized imagery, such as icons (eg, prescription, emergency, secure messaging), to be used to standardize the look of imagery across platforms.

##### Design

**Figure 14:**
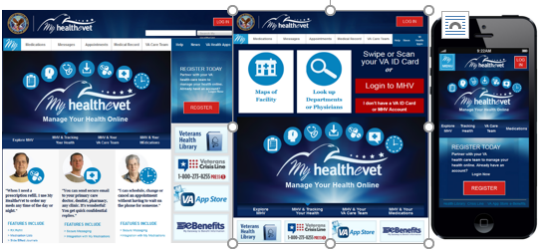
Visual model simulations of VA HIT standardization across various platforms (webpage, VetLink Kiosk, and mobile) based on data from group modeling activity.

Veteran participants preferred a dashboard design for all VA HIT interfaces. The dashboard would be uncluttered, easy to use, and contain universally recognizable icons with large text. Many veteran participants said the look and feel should be based on commonly used software apps. One participant declared the dashboard should look like a car’s dashboard with “everything in one place.” When discussing MHV, participants also preferred to navigate from the homepage to features in one or two mouse clicks. They preferred that important information be centrally located, while news, updates, and other information be located at the bottom of the dashboard or omitted entirely. Standardization and design features captured across HIT platforms were simulated based on participants’ voiced preferences. Figure 14 presents how standardization of esthetics and design features across HIT platforms were simulated based on the group modeling activity.

##### Authentication

Participants preferred the secure nature of the initial in-person authentication currently required to allow veterans to access MHV advanced services such as SM and Blue Button. Those veteran participants that preferred online authentication wanted to provide their social security number and answer security questions to allow a remote but secure process for authenticating access.

#### Delegation and Sharing Information With Community Providers

Participants wanted to securely and electronically share their health care information with community providers and informal caregivers such as their spouses/partners, siblings, parents, or adult children. They appreciated the convenience of delegation. Many veteran participants reported that they already allowed family members to use their secure credentials to access their MHV accounts to help manage their health. Participants felt that they should be able to personalize levels of access to their EHRs and control who has access to different types of health information.

#### Single Sign-On/Federated Credentialing

Participants’ preferences regarding federated credentialing “single sign-on” varied depending on their knowledge of and proficiency in using technology. Commonly, those veteran participants who supported single sign-on explained that they had “password fatigue,” with general difficulty remembering usernames and passwords. While nearly all veteran participants acknowledged the expediency of a single-sign on or third-party credentialing mechanism, high-volume users were less likely to prefer this process, citing concerns about security. Low-volume users were less likely to understand federated credentialing but more likely to prefer it after it had been explained. Participants with security concerns were especially wary of credentialing via social media or private email accounts; however, they generally supported the idea of federated credentialing between government websites due to perceptions of high government Internet security standards.

### Accessing Information and Education About VA HIT

When asked how the VA could improve awareness and use of VA HIT, most participants believed educating veterans about the availability and use of VA technologies is critical. Suggested education methods included peer-to-peer mentoring programs, instructional text in the form of a website or user guide, and instructional videos.

### Pairwise Comparisons

Veteran participants preferred to access MHV resources such as SM, Appointment Reminders, Blue Button, etc, through a mobile app. However, there was uncertainty about the security, accessibility, and usefulness of mobile apps when managing health care. A slim majority of focus group participants preferred to access a VA electronic health resource using an Internet browser. Contrary to veteran participant preferences, EPMs overwhelmingly believed that veterans would prefer to use a mobile app to complete health care management tasks. In general, it was apparent that EPMs had a precise knowledge of which tool was designed for a given task. Differences between EPMs and veteran participant responses appeared to be largely based on EPM expert knowledge of resources, logistics, cost, and workflow issues.

Veteran participants and EPMs agreed that SM is the preferred resource for completing a wide variety of health care management tasks. Veteran participants included SM in their lists of useful comparison resources more often than any other resource, and EPMs frequently ranked SM higher than most other available resources, suggesting that both groups considered it a useful tool for completing a range of health care management tasks even when it was not the most preferred option. Findings indicate that no single HIT solution is acceptable for the full range of health-related tasks, and a full range of options is needed. Preferences can change based on the individual or situation. Preferences among veteran participants and EPMs, and the resources they agreed on are presented in Table 6.

**Table 6:** Pairwise comparison agreement between participant groups.

Task	Veteran participant only	EPM only	Veteran participant and EPM
Communicate with care team		Mobile App	Secure Messaging
Review lab results	Labs and tests	Mobile App Blue Button	Secure Messaging
Research medical information	Blue Button Non-VA websites	Mobile App	Veterans Health Library (general information)
Track vital signs	Non-VA Vitals Tracker	Mobile App	MHV Vitals Tracker
	Telehealth		
Request appointment	Secure Messaging	Mobile App	MHV Appointment Reminders
		Text messaging	
Cancel/ reschedule appointment	MHV Appointment Reminders	Mobile App	Telephone
		Secure Messaging	
Order Rx refill	Secure Messaging	Mobile App	MHV Rx Refill
	MHV Appointment Reminders		
Rx refill notification		Mobile App	Secure Messaging

## Discussion

### Principal Results

The goal of this study was to inform the VA’s vision of an integrated HIT system from the veteran perspective [8,9]. Veteran participants value virtual health care delivery and are invested in having access to care anytime, anywhere [12]. Many of the current systems were designed to address a perceived need or fulfill a policy directive. Thus, the greatest value of this work is the development of veteran-driven high-fidelity modeling simulations and assets that illustrate user needs and expectations when using a HIT system and services to access VA health care services. These are critical contributions to the VA, a “patient-centered” organization that seeks to incorporate “the veteran voice” into all service areas, including HIT.

Focus group findings illustrate the role of VA HIT in self-management of health care and chronic illness. It is logical that veterans with multiple chronic illnesses would rely heavily on technologies that support regular communication with providers (Secure Messaging), facilitate appointments (Appointment Reminders), and help with prescription management (RX Refill). For example, we now understand that tools like Rx Refill are vital to veterans with multiple chronic illnesses because they often manage many medications. Similarly, these findings provide important insights about barriers to use, along with suggestions for improvement. Veteran participants highlighted some of the functional improvements that could be made to help them manage a large volume of prescriptions, such as providing prescription expiration and refill notifications to help them maintain medication compliance.

One major finding of this study reaches beyond the needs of veterans with multiple chronic illnesses. Our participants expressed a strong preference for standardized, integrated, and synchronized user-friendly interface designs. Although standardization has long been an issue of importance to usability and design efforts [13], improvements in standardization across VA HIT resources are needed to optimize effective usage. The participants in this study recognized VA HIT’s lack of visual standardization across platforms as a departure from many of the health management technologies available in the private sector and emphasized that improving the “look” of VA HIT was a critical step toward system modernization and promoting use. In addition, current system navigation and usability issues and concerns about security, back-up systems, and delegation can be successfully enhanced with a human-centered design approach. For example, veteran participants’ preferences suggest that navigation and security issues and issues of standardization may influence the potential for adoption and sustained use. When VA HITs appear and function consistently across platforms, it creates a level of recognition that promotes comfort. This could also impact the uptake of newly released VA HIT. It is likely that if new HITs have the same look, feel, and general functionality as other HIT, veterans will have fewer problems learning to use new systems and apps. There was evidence to suggest education on features such as federated credentialing, single sign-on, and associated security issues is needed to promote the acceptance of these features. To ensure veterans are aware of and know how to use VA HIT, the veterans in our study suggested just-in-time marketing and education about how to access and use VA HIT resources.

The pairwise comparison focus group activity provided a unique way of discovering user preferences for use of VA technology platforms. Veteran participants expressed specific preferences for the platforms they wanted to use to accomplish specific tasks, its sense of urgency, and other situational contexts. Veteran participants and expert panel members agreed that a full range of options is needed, noting preferences can change based on the individual, the specific task, or the situation. In general, both veteran participants and expert panel members considered SM the most preferred resource. A slim majority of veteran participants preferred to access electronic health resources such as SM, Appointment Reminders, or Blue Button, using an Internet browser rather than a mobile app, in contrast to the belief of expert panel members who overwhelmingly believed that patients would prefer to use a mobile app. Veteran participants who did want to access resources through mobile apps expressed uncertainty about security, accessibility, and usefulness. Differences between veteran participant and expert panel member perspectives may be the result of panel member’s knowledge of logistics, cost, and workflow issues, as well as insights about future technology (mobile apps).

Future research should inform VA’s vision for an integrated HIT system to include front-end veteran user experiences and outcomes. Specifically, research should evaluate best practices for supporting patients’ proactive and integrated use of VA HIT systems. In addition to assessing front-end veteran user experiences, veteran data also indicate that organization level research is needed to identify large-scale infrastructural consequences relevant to the supply and demand of the growing VA patient population. This research should assess the dynamic interaction of patient-provider electronic communication, and provider and team experiences, including workload and workflow, in order to ensure that the back-end systems and processes supporting the front-end veteran experience are operating effectively. Finally, system preferences such as single sign-on and delegation merit further investigation to better understand the feasibility, acceptability, and usefulness of these features within the current and evolving VA HIT system across traditional (eg, personal computers) and emerging (eg, mobile) technologies. Delegation has become increasingly important as the VA places more emphasis on engaging with community care providers and family care givers. Provision of comprehensive and consistent veteran health care rests on the veteran’s ability to securely and easily delegate access to medical records and virtual health services.

### Comparison With Prior Work

This work builds on previous work exploring user experiences on individual HIT platforms and tools within and beyond the VA. However, to our knowledge this is the first study to look at user experience across an enterprise-wide system of VA HIT platforms and tools. The unique contribution of this work is its comprehensive approach to looking at currently available VA HIT capacity and emerging functionality. As such, the modeling simulations produced in this work are veteran driven and can inform ongoing VA HIT redesign initiatives.

### Limitations

Although this study underscored veteran preferences for using HIT and offered their recommendations for system improvements, it had some limitations. First, the study reports findings from two specific VA facilities. While participants were a representative purposively sampled group [14], additional insights may be gained by expanding this assessment to other VA facilities and veteran populations. Second, findings are primarily relevant to VA HIT systems and technologies but may be useful for the development and redesign of other tethered HIT systems. Third, current technological infrastructure capacity was not a primary focus and thus some desired changes may not yet be technically possible. Fourth, we purposively recruited participants who were invested users of two or more platforms; we may have missed valuable data that may have represented non-invested users. Finally, we included veterans with chronic conditions because they are more likely to leverage electronic resources to manage their health care, as such, we may have missed valuable data that may represent healthier participants [15,16].

### Conclusions

This is one of the few published studies to aid in the development of an integrated system of patient-facing HIT resources within a large health care system. The findings from this study have already had a direct impact on the incremental redesign of the My HealtheVet patient portal and the prioritization of approaches that provide integration between VA HIT platforms. Future research can inform the ongoing development of VA’s integrated HIT system, to include front-end patient user experiences and back-end workload and workflow. Future work should evaluate best practices for supporting consumers’ proactive and integrated use of VA HIT systems. Though this research lends itself to recommendations for future research, our aim in completing this work was to inform a user-centric perspective to assist ongoing development, redesign, and research efforts. These assets were developed from a veteran-centric perspective to support the use of VA’s dedicated resources to materialize the findings in ongoing VA HIT redesign efforts. Organizations beyond the VA can benefit from using a similar approach and may discover the findings useful in designing human-centered HIT systems.

## Abbreviations

EHR: electronic health record
EPM: expert panel members
HIT: health information technology
MHV: My HealtheVet
SM: Secure Messaging
VA: Department of Veterans Affairs
